# Assessment of cardiac dyssynchrony: a comparison of velocity encoded imaging and feature tracking analysis

**DOI:** 10.1186/1532-429X-15-S1-M11

**Published:** 2013-01-30

**Authors:** Daniel Kuetting, Jonas Doerner, Alois M Sprinkart, Claas P Naehle, Hans H Schild, Daniel Thomas

**Affiliations:** 1 Radiology, University of Bonn, Bonn, Germany

## Background

Cardiac dyssynchrony can be found in up to 99% of patients suffering from chronic heart failure. MRI VENC (velocity encoded imaging), along with TDI, have been shown to accurately assess myocardial dyssynchrony. Long acquisition times of VENC and a high inter-examiner variability of TDI have prohibited a broad clinical application of these modalities thus far. Recently a new method utilizing feature tracking was introduced which allows to analyze strain, respectively velocities, in standard SSFP cine images.

This study sought to assess the feasibility of evaluating dyssynchrony in standard SSFP cine images with the use of feature tracking analysis.

## Methods

Velocity encoded MRI was performed in 2 groups, 13 healthy control subjects (group A) and 6 dyssynchronous patients pre- CRT (cardiac resynchronization therapy) (group B). Additionally, high temporal resolution SSFP cine images were acquired. Quantitative analysis of dyssynchrony in VENC encoded MR-images was performed using Segment 1.9 software package (Medviso AB). Venc imaging was performed in the four-chamber orientation. Velocity was encoded with a velocity sensitivity of 20 cm/s. Additionally SSFP-cine images with the same temporal resolution were acquired in the four chamber orientation. Velocity was measured in the basal segments of the lateral free wall and the septum. The analysis of dyssynchrony in SSFP cine-images was performed with 2D CPA MR (TomTec Imaging Systems, Germany).

## Results

Intraventricular dyssynchrony was not observed in group A (mean dyssynchrony 12.3 ± 9.6 ms using VENC analysis, mean dyssynchrony 10.6 ± 9.6 ms on feature tracking (p=NS)). Group B displayed a mean intraventricular dyssynchrony of 77.9 ± 29.5ms with VENC analysis, 74.8 ± 26.5ms with feature tracking respectively (p=NS). The feature tracking method reliably discriminated between dyssynchrony and physiologically timed contraction in all cases. A good correlation (Pearson 0,9673 P<0,0001) and agreement (mean difference 2.7 with a 95% CI of -1,7875 to 7,5057) were found between VENC MR imaging and feature tracking.

## Conclusions

Preliminary results suggest that feature tracking analysis of standard SSFP-cine images can yield equivalent information on intraventricular dyssynchrony to VENC imaging. This would permit an assessment of dyssynchrony in a clinical setting without having to acquire time consuming VENC images. Future studies are needed to further validate these findings.

## Funding

There was no funding source for this study.

**Figure 1:**
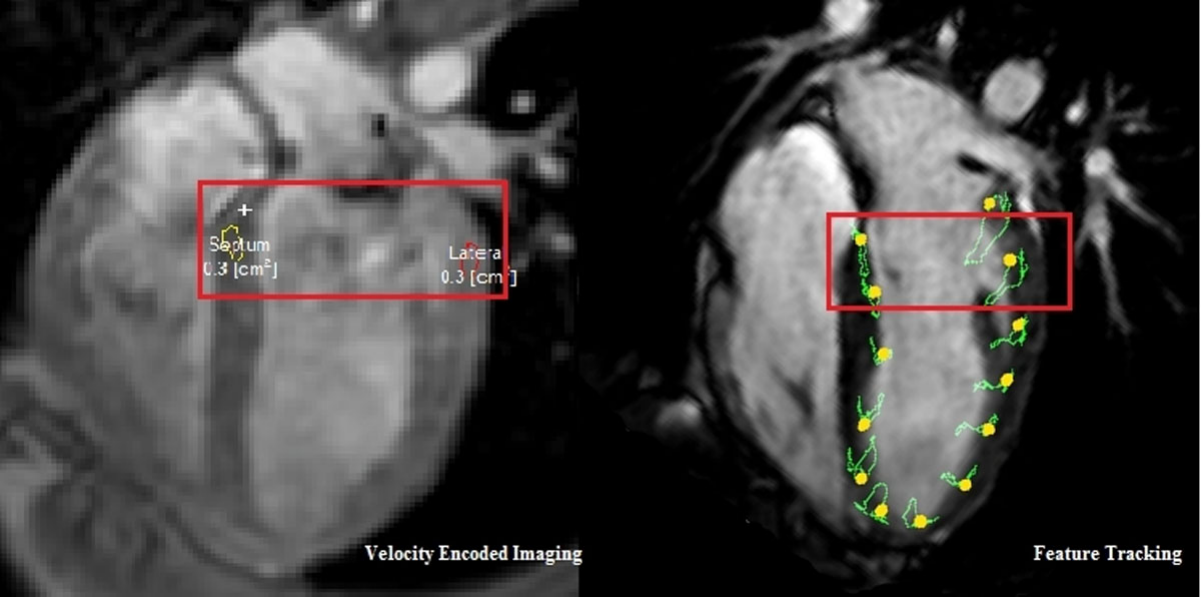
Defined regions of velocity measurement. (left image: Velocity Encoded Imaging; right image: Feature Tracking)

**Figure 2:**
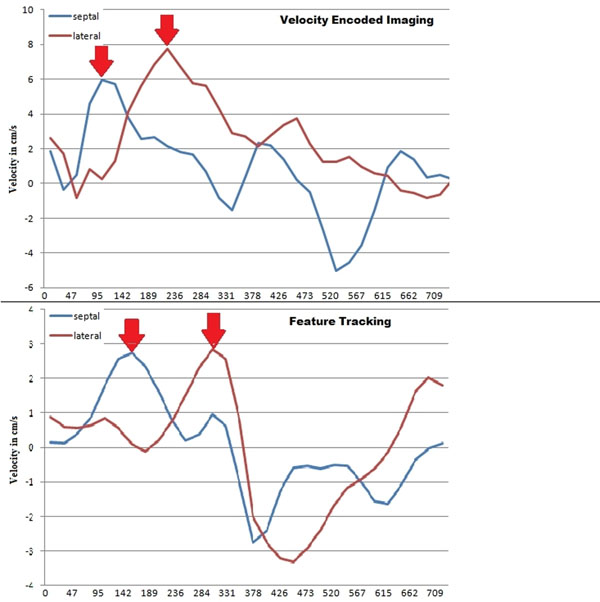
Results of Velocity Encoded Imaging (upper graph) and Feature Tracking (lower graph) analysis of a dyssynchronous patient displaying extensive septal-to-lateral-delay.

